# New exceptionally preserved arthropod from the Furongian of Canada

**DOI:** 10.1186/s12915-026-02617-4

**Published:** 2026-05-28

**Authors:** Russell D. C. Bicknell, Julien Kimmig, Aaron Goodman, Thomas Turner, Patrick M. Smith

**Affiliations:** 1 College of Science and Engineering, Flinders University, Adelaide, SA Australia; 2 Division of Paleontology (Invertebrates), American Museum of Natural History, New York, NY USA; 3 Institute of Applied Geosciences, Karlsruhe Institute of Technology (KIT), Adenauerring 20B, 76131 Karlsruhe, Germany; 4 Paläontologie, Staatliches Museum Für Naturkunde Karlsruhe, Karlsruhe, 76133 Germany; 5 School of Geology & Geological Engineering, The Harold Hamm, University of North Dakota, Grand Forks, ND 58202 USA; 6 Department of Entomology, University of Illinois Urbana Champaign, Urbana, Il 61801 USA; 7 Division of Invertebrate Zoology, American Museum of Natural History, New York City, NY USA; 8 Graduate Center, City University of New York, New York City, NY USA; 9 Geological Survey of New South Wales, Division of Mining, Exploration and Geoscience, Department of Regional New South Wales, Londonderry, NSW Australia; 10 Department of Biological Sciences, Macquarie University, Sydney, NSW Australia

**Keywords:** Corcoraniidae, Chelicerata, Cambrian, Euarthropoda, Exceptional preservation

## Abstract

**Background:**

Corcoraniidae is a rare and understudied family of lower Paleozoic euarthropods that are important for understanding early chelicerate evolution. However, the diversity, morphology, and distribution of this group remain poorly resolved, particularly during the late Cambrian.

**Results:**

Here, we describe a new corcoraniid from the late Cambrian (Furongian) Rivière-du-Loup Formation of Quebec, Canada. The distinctive morphology of this specimen warrants erection of a new genus and species, *Magnicornaspis garwoodi* gen. et sp. nov. This new taxon expands the morphological disparity in Corcoraniidae and suggests that a shift in location of large cephalic spines arose within older members of the clade. The presence of a corcoraniid within the Rivière-du-Loup Formation provides a crucial data point within the “Furongian Gap” suggesting that perceived biodiversity declines may be an artifact of sampling bias rather than anoxia-driven extinction.

**Conclusions:**

This material highlights the potential for exceptional soft-bodied preservation within deeper-water lithofacies and warrants renewed investigation of the Rivière-du-Loup Formation. The deposit has a high probability of representing a new Furongian Konservat-Lagerstätte that will present important insight into late Cambrian biodiversity and the evolutionary history of early arthropods.

**Supplementary Information:**

The online version contains supplementary material available at 10.1186/s12915-026-02617-4.

## Background

Corcoraniidae is an understudied group of lower Paleozoic euarthropods [1]. The phylogenetic position of these arthropods has varied [2, 3], with the current interpretation suggesting that the group are basal chelicerates [3, 4]. Corcoraniidae consists of unbiomineralised euarthropods that have comparably sized cephalic and pygidial shields, along with a seven-segmented thorax [1, 5] and has a fossil record spanning from the Cambrian through to the Lower Ordovician (Table 1; [1]). Despite the rarity and underexamined nature of corcoraniids, the family has presented an exceptional wealth of anatomical information [2], including evidence for ancient central nervous systems [3, 4], as well as insight into changes of these structures within Arthropoda [2]. As such, corcoraniids are an important lineage for understanding chelicerate evolution.

**Table 1:** Summary of documented corcoraniids. Observations ordered temporally and then alphabetically by genus and species

Observation	Formation, Locality	Series (Age)	Reference
**Cambrian**			
* Mollisonia* sp.	Shuijingtuo Formation, China	Cambrian Series 2 (Stage 3)	Fu, et al. [6]
* Mollisonia* sp.	Balang Formation, China	Cambrian Series 2 (Stage 4)	Zeng, et al. [7]
*Mollisonia sinica* Zhang et al., 2002 [8]	Kaili Formation, China	Miaolingian (Wuliuan)	Yuanlong, et al. [9], Zhang, et al. [8]
*Mollisonia* *plenovenatrix* Aria and Caron, 2019 [2]	Burgess Shale Formation, British Columbia, Canada	Miaolingian (Wuliuan)	Aria and Caron [2]
*Mollisonia symmetrica* Walcott, 1912 [10]	Spence Shale Member, Langston Formation, Utah, USA	Miaolingian (Wuliuan)	Briggs, et al. [11]
*Mollisonia symmetrica*	Walcott Quarry Shale Member, Burgess Shale Formation, British Columbia, Canada	Miaolingian (Wuliuan)	Ortega-Hernández, et al. [3], Walcott [10], Simonetta and Delle Cave [12], Størmer in Moore and Teichert [13], Simonetta [14]
*Thelxiope palaeothalassia* Simonetta and Delle Cave, 1975 [12]	Burgess Shale Formation, British Columbia, Canada	Miaolingian (Wuliuan)	Lerosey-Aubril, et al. [1], Simonetta and Delle Cave [12]
*Thelxiope cf. T. palaeothalassia*	Spence Shale Member, Langston Formation, Utah, USA	Miaolingian (Wuliuan)	Kimmig, et al. [15]
*Mollisonia symmetrica*	Wheeler Formation, Utah, USA	Miaolingian (Drumian)	Lerosey-Aubril, et al. [5], Gunther and Gunther [16], Robison [17]
*Mollisonia symmetrica*	Linyi Lagerstätte, Zhangxia Formation, China	Miaolingian (Drumian)	Sun, et al. [18]
*Thelxiope holmani* Lerosey-Aubril et al., 2020 [1]	Wheeler Formation, Utah, USA	Miaolingian (Drumian)	Lerosey-Aubril, et al. [1]
*Thelxiope spinosa* (Conway Morris & Robison, 1988) [7]	Wheeler Formation, Utah, USA	Miaolingian (Drumian)	Lerosey-Aubril, et al. [1], Conway Morris and Robison [19], Robison [17], Robison, et al. [20]
*Thelxiope spinosa*	Linyi Lagerstätte, Zhangxia Formation, China	Miaolingian (Drumian)	Sun, et al. [18]
*Thelxiope tangi* Sun et al., 2022 [18]	Linyi Lagerstätte, Zhangxia Formation, China	Miaolingian (Drumian)	Sun, et al. [18]
*Magnicornaspis garwoodi* gen. et sp. nov	Rivière-du-Loup Formation, Québec, Canada	Furongian (Stage 10)	This article
**Ordovician**			
*Corcorania trispinosa* Jell, 1980 [21]	Castlemaine Group, Victoria, Australia	Early Ordovician (Tremadocian)	Jell [21]
Mollisoniid-like arthropod	Dol-cyn-Afon Formation, Wales, UK	Early Ordovician (Tremadocian)	Botting, et al. [22]
*Thelxiope* sp.	Fezouata Shale, Ternata Plain, Morocco	Early Ordovician (Tremadocian)	Lerosey-Aubril, et al. [1], Van Roy, et al. [23]
*Mollisonia* sp.	Bøggild Formation, North Greenland	Early Ordovician (Floian)	Peel, et al. [24]

A key direction towards uncovering the diversity of this group involves the identification of new specimens [1]. To address this need, we present a new example of a corcoraniid from the late Cambrian (Furongian) of Quebec. The material is broadly comparable to forms from Australia [21], but shows sufficient morphological differentiation to warrant a new genus. In addition to presenting this new taxon, we explore its preservation and the paleogeography of the group. Examination of this new material also highlights the possibility of a new late Cambrian exceptional preservation site, expanding the rapidly developing record of similarly aged deposits [22–27].

### Geological context

**Fig. 1:**
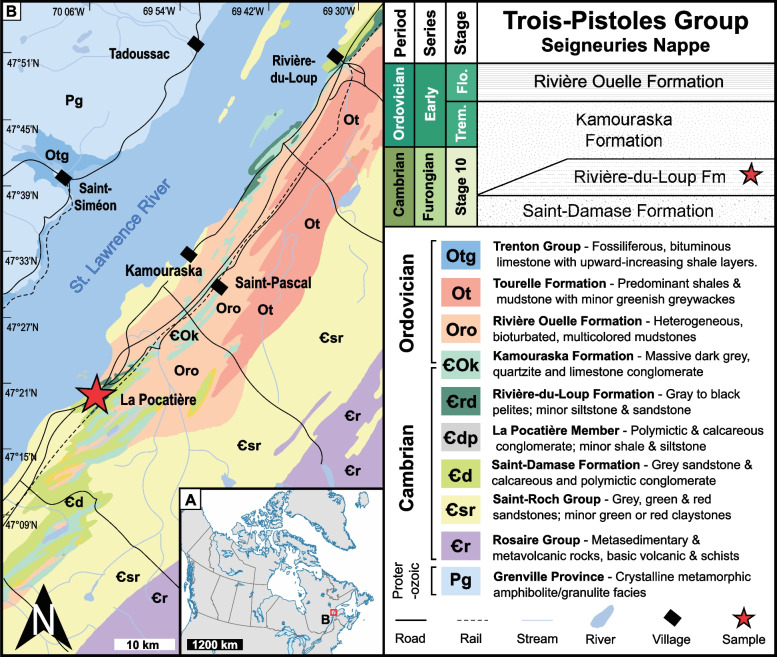
**A** Map of Canada, showing sample site near Quebec. **B** Close-up on specimen locations (star) in the La-Pocatière region with local bedrock geology. Top right, stratigraphic column for the Seigneuries Nappe with the Trois-Pistoles Group plotted against time. Abbreviations: Flo: Floian. Trem: Tremadocian)

USNM PAL 801575 was collected by C. Hubert in 1962 during geological mapping of the Sainte-Anne-de-la-Pocatière region. Museum records and accompanying field labels indicate the material was recovered from black shales at a locality near the Institut de technologie agroalimentaire du Québec (ITAQ), Campus de La Pocatière, in Sainte-Anne-de-la-Pocatière, Québec. Hubert [28] originally assigned this strata and specimen to the Sainte-Anne Member—the uppermost unit of the Saint-Damase Formation within the Trois-Pistoles Group (Fig. 1). The region has a long history of study by Logan [29, 30], Richardson [31], and McGerrigle [32], with early interpretations attributing the lithologies to the Quebec Group or the Lévis and Sillery formations. These rocks are part of the Seigneuries Nappe, a structural unit extending northeast to the Rimouski region.

While Hubert [28] initially estimated the thickness of the Saint-Anne Member to be approximately 365 m near its type area, subsequent stratigraphic revisions [33] reassigned the pelitic levels at the top of the Saint-Damase Member to a new unit, the Rivière-du-Loup Formation [34] (Fig. 1). In the L’Islet-Kamouraska sector, this formation constitutes a relatively thin pelitic assemblage of approximately 15 m [28, 33–36]. The matrix of USNM PAL 801575 is lithologically indistinguishable from these beds, consisting primarily of medium to dark gray pelities (Hubert’s “black shale”) interstratified with gray siltstone (20%) and sandstone (5%). The pelities range from 1–10 cm in thickness, while siltstone and sandstone beds vary between 0.5–10 cm [33, 34].

The depositional environment of the Rivière-du-Loup Formation is interpreted as a fine-grained siliciclastic slope record [28, 34, 36, 37]. This setting represents a transition from the higher-energy, cyclic turbidite systems of the underlying Saint-Damase Formation, which is typified by thick, graded feldspathic arenites and limestone conglomerates. In contrast, the Rivière-du-Loup assemblage reflects a more tranquil, distal slope environment dominated by the settling of fine-grained muds from suspension [37]. The presence of parallel, wavy, and convolute laminae in the siltstone and sandstone beds suggests that the environment was periodically influenced by low-density turbidity currents [33, 34].

Occurrence of dendroid graptolites, specifically *Callograptus* Hall, 1865 [38] (previously assigned to *Dictyonema?* Hall, 1851 [39]), originally suggested a late Cambrian (Furongian) to Early Ordovician (Tremadocian) age for the unit [28, 40]. This age assignment was later corroborated by age-equivalent acritarchs and scolecodonts reported from northern exposures of the formation [37, 41, 42]. Biostratigraphically, the Rivière-du-Loup Formation more broadly is constrained by the underlying La Pocatière Member of the Saint-Damase Formation and the overlying Kamouraska Formation. Both units contain basal conglomerates with allochthonous trilobite faunas of early Late Cambrian (Regional Stage: Dresbachian) and latest Cambrian (Regional Stage: Trempealeauan) age, respectively [17]. This interval aligns well with previous interpretations of the depositional history of the Rivière-du-Loup Formation, which place it during a sea-level highstand interpreted as “Grand Cycle C” (of James and Stevens [43]; James, et al. [44], and Lavoie, et al. [37] for correlation). This places the unit within the regional late Steptoan or Sunwaptan stages, equivalent to the global Stage 10, Furongian [45].

### Systematic paleontology

*Phylum* EUARTHROPODA Lankester, 1904 [46]

*Subphylum* CHELICERATA Heymons, 1901 [47]

*Order* MOLLISONIIDA Lerosey-Aubril et al., 2020a [5]

*Family* CORCORANIIDAE Jell, 1980 [21]

(= MOLLISONIIDAE Lerosey-Aubril et al., 2020a [5]).

*Diagnosis*: Unmodified from Lerosey-Aubril, et al. ([5], p. 518) “Mollisoniid chelicerates exhibiting well-defined eye notches on the cephalon and a thorax composed of seven freely articulating tergites.”

*Type-genus*: *Corcorania* Jell, 1980 [21]

*Other genera included*: *Magnicornaspis* gen. et sp. nov.; *Mollisonia* Walcott, 1912 [10]; *Thelxiope* Simonetta and Delle Cave, 1975 [12].

*Occurrences*: Cambrian—Shuijingtuo Formation, China (Cambrian Series 2, Stage 3) [6]; Kaili Formation, China (Miaolingian, Wuliuan) [8, 9]; Burgess Shale Formation, British Columbia, Canada (Miaolingian, Wuliuan) [1, 3, 10, 12–14]; Langston Formation, Utah, USA (Miaolingian, Wuliuan) [11, 15]; Wheeler Formation, Utah, USA (Miaolingian, Drumian) [1, 16, 17]; Zhangxia Formation, China (Miaolingian, Drumian) [18]; Rivière-du-Loup Formation, Québec, Canada (Furongian, Stage 10). Ordovician—Castlemaine Group, Victoria, Australia (Early Ordovician, Tremadocian) [21]; Dol-cyn-Afon Formation, Wales, UK (Early Ordovician, Tremadocian) [22]; Fezouata Shale, Ternata Plain, Morocco (Early Ordovician, Tremadocian) [1, 23]; Bøggild Formation, North Greenland (Early Ordovician, Floian) [24].

*Remarks*: During review of this manuscript, an important nomenclatural issue concerning the family-level designation was raised by a reviewer. The name Corcoraniidae proposed in Jell [21] was established in association with the description and diagnosis of *Corcorania*. In doing so, Jell [21] had satisfied the following—“A combined description or definition of a new nominal genus or subgenus and a single included new nominal species, if marked by “gen. nov., sp. nov.” or an equivalent expression, is deemed to confer availability on each name” ([48], Article 13.5). This predates the treatment in Lerosey-Aubril, et al. [5] who noted that Mollisoniidae had previously been introduced without diagnosis or definition by Simonetta and Delle Cave [12]. Because *Corcorania* falls within the group otherwise referred to as Mollisoniidae, Corcoraniidae has priority, rendering Mollisoniidae the junior synonym. We therefore adopt Corcoraniidae here. Aside from this nomenclatural correction, the systematic framework of Lerosey-Aubril, et al. [5], remains unchanged and was otherwise rigorously developed.

*Subfamily* CORCORANIINAE Jell, 1980 [21]

*Diagnosis*: Corcoraniid arthropod with hypertrophied spines, anteriorly directed cephalic spines, thoracic tergites lacking terminal or hypertrophied dorsal spines, and sub-triangular pygidial structures lacking pygidial ridges.

*Type-genus*: *Corcorania* Jell, 1980 [21]

*Other genera included*: *Magnicornaspis* gen. nov.

*Occurrences*: Rivière-du-Loup Formation, Québec, Canada (Furongian, Stage 10); Castlemaine Group, Victoria, Australia (Early Ordovician, Tremadocian) [21].

*Remarks*: Lerosey-Aubril, et al. [5] presented a thorough reassessment of *Mollisonia* and erected Mollisoniidae (= Corcoraniidae) to encompass the genus and related taxa. In this assessment, the authors noted that Corcoraniidae of Jell [21] should likely be considered a subfamily (Corcoraniinae), reflecting comparable overall morphology, particularly the seven-segmented thorax, while also acknowledging distinctive characters. However, no formal diagnosis of the subfamily was provided. Here, we address this point and provide an updated diagnosis for Corcoraniinae.

*Genus*: *Magnicornaspis* gen. nov.

*Etymology*: From Latin *magnus* (large), *cornu* (horn), and *aspis* (shield).

*Diagnosis*: As for species.

**Fig. 2:**
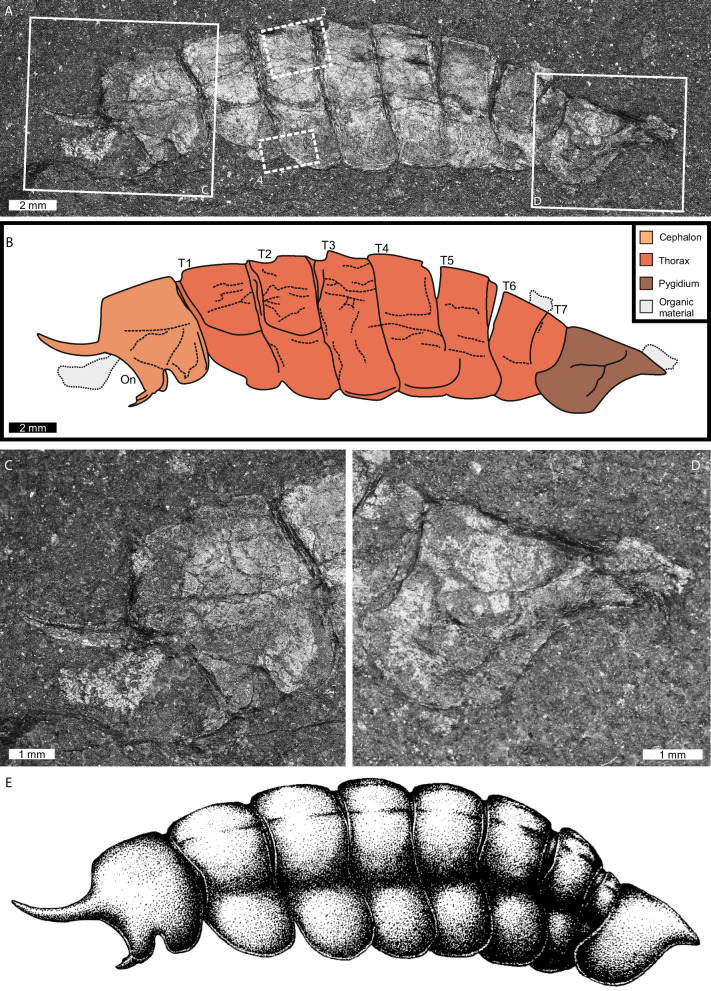
Depiction of *Magnicornaspis garwoodi* gen. et sp. nov*.* USNM PAL 801575. **A** Complete specimen. Boxes with solid lines show close ups **C** and **D**. Boxes with dotted lines show areas that were imaged using SEM and EDS maps in Figs. 3 and 4. **B** Line drawing of specimen. **C** Close up of cephalon showing hypertrophied spine. **D** Close up of pygidium, showing terminal spine. **E** Reconstruction of *Magnicornaspis garwoodi* gen. et sp. nov*.* (**A**, **C**, **D**) under flash photography. Abbreviations: On: optical notch. T: thoracic tergite. Numbers (1–7) indicate thoracic tergite numbers

*Species*: *Magnicornaspis garwoodi* gen. et sp. nov. (Fig. 2).

*Etymology*: The specific name was selected in recognition of Russell Garwood who has committed his career to documenting chelicerate evolution.

*Diagnosis*: Corcoraniid with two anteriorly directed cephalic spines, thoracic tergites lacking dorsal spines, and a sub-triangular pygidium lacking hypertrophied spines.

*Holotype*: USNM PAL 801575.

*Formation, locality, and age*: Rivière-du-Loup Formation unspecified locality near the Institut de technologie agroalimentaire du Québec (ITAQ)—Campus de La Pocatière, in Sainte-Anne-de—la-Pocatière, Québec (Furongian, Cambrian).

*Preservation*: Specimen preserved as a laterally oriented compression fossil in black shale, lacking topographic relief.

*Description*: Cephalic shield 4.42 mm long and 4.99 mm wide, with rounded tergopleural regions (Fig. 2A–C). Anterior margin weakly convex, with a possible ocular notch. One large spine projects from anteriormost region, with a smaller spine arising from ventral portion of anterior margin. Ventral margin moderately convex with a distinct U-shaped indentation. Posterior margin straight and lacking spines. Possible degraded organic material occurs at anteriormost cephalic region. Thorax 16.82 mm long, composed of seven articulated tergites (Table 2; T1–T7), with tergites broadly similar in size, except T7, which is thinner and shorter. All tergites lack terminal and dorsal spines. Pygidium 5.29 mm long and 3.69 mm wide, sub-triangular, bearing a single terminal spine (Fig. 2D). No sagittal or pygidial ridges observed.

**Table 2:** Summary of thoracic dimensions for USNM PAL 801575. * indicates partial measurement

Measurement	Tergite 1	Tergite 2	Tergite 3	Tergite 4	Tergite 5	Tergite 6	Tergite 7
Width (mm)	5.76	6.26	6.61	6.48	5.65	4.61	2.37*
Length (mm)	3.22	2.67	2.72	2.57	2.21	1.94	1.49

#### Remarks

**Fig. 3:**
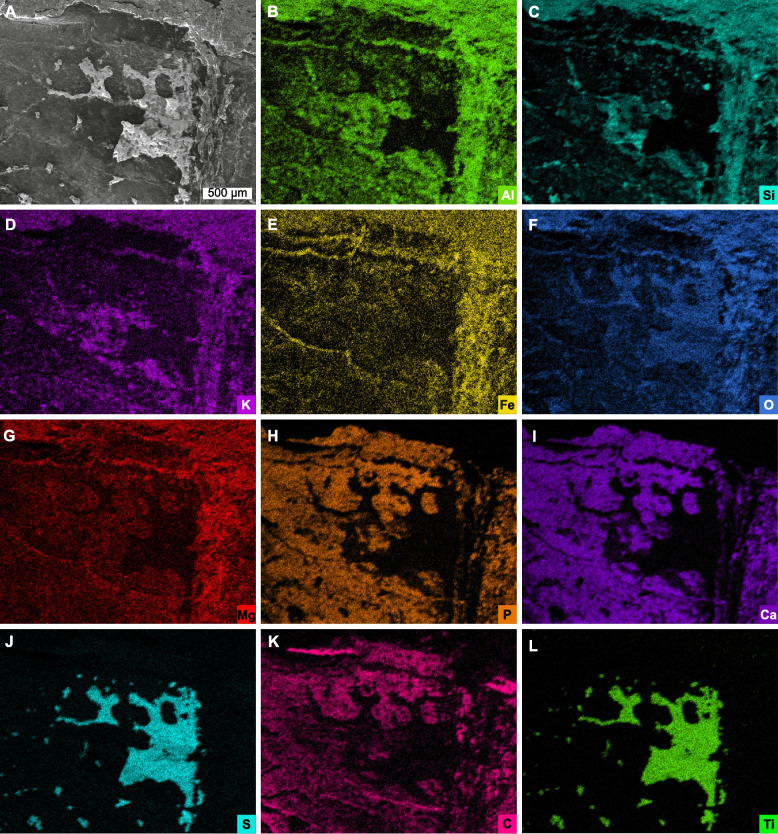
SEM backscatter image and EDS elemental maps of thoracic dorsum; area indicated in Fig. 2A. **A** Backscatter image of dorsal carapace. **B–L** Elemental maps of aluminum, silicon, potassium, iron, oxygen, magnesium, phosphorus, calcium, sulfur, carbon, and titanium, respectively

The presence of seven thoracic tergites and an approximately isopygous condition supports placement within Corcoraniidae (Fig. 2E). The absence of hypertrophied dorsal spines on the thorax and pygidium excludes assignment to *Thelxiope* [1, 15]. The presence of prominent anterior cephalic spines excludes our material from *Mollisonia* [5, 10, 11]*.* These features, combined with pygidial morphology, and a reduced seventh thoracic tergite, support affinity with *Corcorania* ([21], Fig. 3D, E). However, *Corcorania* exhibits three cephalic spines, contrasting with the two spines observed here. The dorsal-most cephalic spine is reduced in *Corcorania* and this spine is hypertrophied in the material we considered here. Finally, *Corcorania* possesses lateral pygidial projections that are not observed in the new taxon. These distinctions justify erection of a new genus and species.

## Results

### Elemental composition

**Fig. 4:**
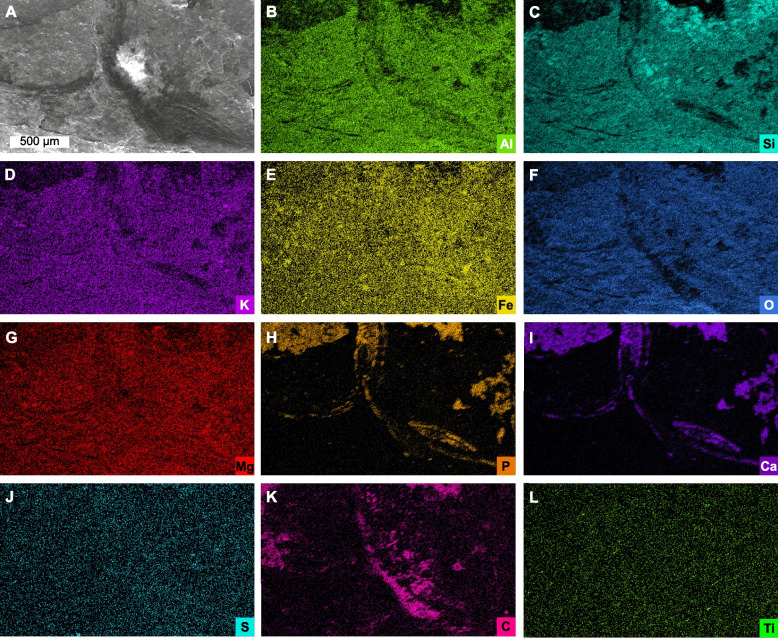
SEM backscatter image and EDS elemental maps of ventral thoracic region; area indicated in Fig. 2A. **A** Backscatter image of ventral thoracic region. **B–L** Elemental maps of aluminum, silicon, potassium, iron, oxygen, magnesium, phosphorus, calcium, sulfur, carbon, and titanium, respectively

The exoskeleton shows enrichment of calcium, phosphorus, carbon, and sulfur (Figs. 3H–K and 4H, I, K), with lower concentrations of potassium, aluminum, silicon, and oxygen (Figs. 3B–D, F and 4B–D, F). Trace amounts of iron, magnesium, and titanium (likely barium, see Discussion) occur proximal to the rock matrix (Fig. 3E, G, L).

### Paleobiogeography

**Fig. 5:**
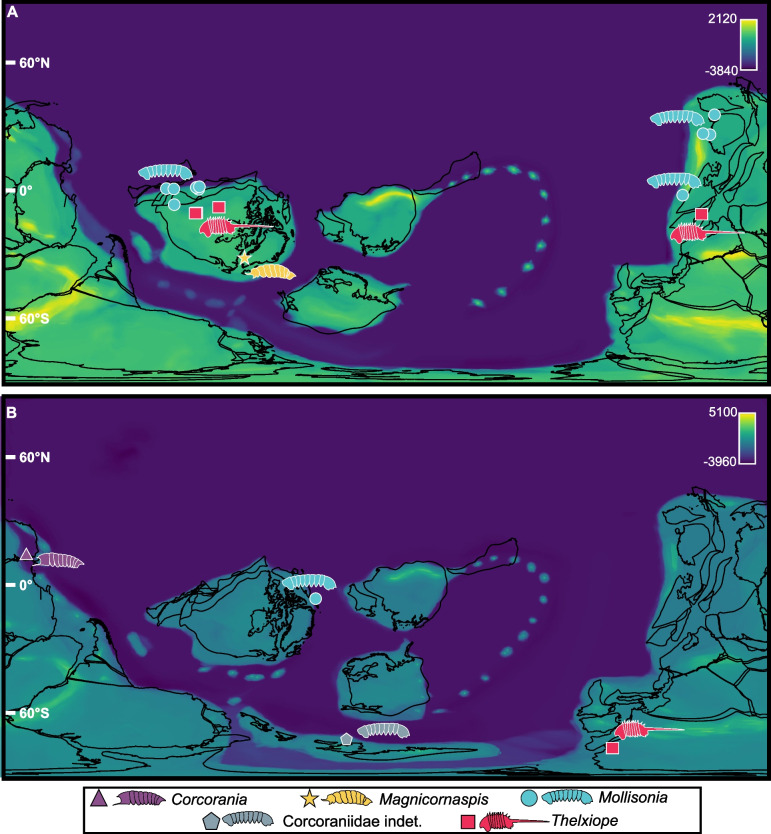
Corcoraniid localities from the Cambrian to Ordovician. **A** Cambrian observations, including new specimen. **B** Ordovician observations. Paleo-elevational maps were acquired using Scotese and Wright [49] and PBDB data

Corcoraniid geographic distributions shift between the Cambrian and the Ordovician (Fig. 5). Cambrian corcoraniids are predominantly distributed along the northern Laurentia and Gondwana coasts at mid-latitudes (~ 0–30°), generally occupying open-marine, subtidal, and deep-water environments. In the Ordovician, corcoraniids persist in northern Laurentia and expand into southern Laurentia and southeastern Gondwana. The genus *Corcorania* occurs in western Gondwana, while the newly described genus *Magnicornaspis* is restricted to southern Baltica (Fig. 5A, B). Depositional environments remain consistent between the two geologic periods.

Estimated bathymetric values range from 3199–38 m below sea level. Cambrian taxa occur between 727–38 m below sea level, whereas Ordovician taxa range from 3199–326 m below sea level. Our newly described taxon *Magnicornaspis* possessed the shallowest bathymetric reading of 38.5 m. Two paleorotated occurrences yielded positive bathymetric values, corresponding to an undescribed species of *Thelxiope*, and an unidentified corcoraniid species. These anomalous values most likely reflect the coarse resolution of the PaleoDEMs.

## Discussion

### Biology of Corcoraniids

The new corcoraniid presented here highlights an important shift in morphology between Cambrian and Ordovician representatives of the group. Until now, Cambrian taxa either show an overall lack of spinose morphologies [3, 10, 12] or exhibit a marked increase in spinosity along the posterior cephalon, dorsal thoracic region, and pygidium [1, 5]. In the Ordovician, some forms display reduced spinosity [1, 23, 24], whereas others show a clear shift towards increased spine development along the anterior cephalon [21]. The presence of a corcoraniid with hypertrophied cephalic spines in the Furongian evidences that these different forms had a Cambrian origin. Furthermore, these patterns suggest a transition towards different defensive strategies within Corcoraniidae.

Although the morphology of Corcoraniidae is well documented within the literature [1–3], there is less information about their ecology. Taxonomic notes have suggested they functioned as benthic micropredators with wide niches that facilitated a worldwide diversification alongside nektobenthic forms [2]. Our paleogeographic maps support this claim, as their distribution ranges throughout shallow seaways, suggesting minimal range contraction. Bathymetric readings for *Mollisonia* ranged from 160 to 752 m below sea level, *Thelxiope* ranged from 0 to 727 m, suggesting a larger bathymetric niche range for *Thelxiope*. The observation of *Corcorania trispinosa* indicated a depth of 3199 m below sea level, suggesting a deep-water preference for Australian forms [21]. *Magnicornaspis* possessed the shallowest bathymetric range (38.5 m), suggesting it may have been a shallow water specialist, ecologically distinct from the other forms. This value also reflects the coarseness of spatial grain in the DEMs, especially as transitions from coastal to deep-water conditions are commonly represented by a few pixels [50]. Nonetheless, depositional environment categories within the Paleobiological Database (PBDB) (“environment” category), supports our findings as most specimens were deposited in deep subtidal ramp, offshore shelf, or indeterminate deep-water or marine settings.

### Taphonomy and preservation

Two elemental maps of the specimen were generated (Figs. 3 and 4), one of the dorsal part of the second tergite (Fig. 3) and one of two ventral parts of the first and second tergites (Fig. 4) and their neighboring sediment. The maps showed that the specimen has a mode of preservation comparable to soft-bodied fossils from Cambrian Konservat-Lagerstätten [51], showing precipitation of phosphate and maturation of carbonaceous remains to kerogen. The continuous presence of calcium and phosphorus in the specimen evidences predominant preservation through phosphatization. This process has been interpreted as diagenetic for early Paleozoic arthropods [52–54], but potential primary calcium phosphate in fossil arthropods has also been discussed [55–58]. In addition, the few studies of living chelicerates appear to show little to no phosphorus present in the exoskeleton [59, 60]. However, Bicknell et al. [56] discussed the issue that fossil crustacean fossils are often preserved as calcium phosphate, whereas studies on modern crustaceans show that calcium phosphate is typically low in concentrations and spatially restricted [61]. In this framework, crustaceans are highlighted as either having incorporated more calcium phosphate into their exoskeletons, or that cuticle is predisposed to diagenetic replacement by calcium phosphate [56].

The fossil also shows carbon enrichment (Figs. 3K and 4K). Carbonate substitution in biological apatite is well documented [62], and significant parts of the carbon maps show a direct spatial overlap with the elemental maps of calcium and phosphorus, suggesting that carbonate substitution is the carbon source. However, in those areas where the specimen is only preserved as carbon, we propose that this represents maturation of carbonaceous remains to kerogen [54, 56].

The dorsal tergite also has bladed crystals composed of titanium and sulfur. This mineralization on the fossil surface as can be seen in the SEM micrograph (Fig. 3A) and likely represents barite mineralization. The apparent presence of titanium is likely an instrumental artifact, owing to the overlap of the barium Lα and titanium Kα X-ray energies (e.g., [54]). While barite can replace soft-tissues [54, 63], the barite in *Magnicornaspis garwoodi* gen. et sp. nov. is not directly replacing or associated with fossil material. Instead, it encrusts the fossil and is therefore interpreted as secondary. Similar encrusting barite crystals have been observed in *Sphenothallus* Hall, 1847 [64] from the lower Cambrian of South China [65].

The preservation of the specimen as carbon-calcium phosphate and the interpreted soft-tissues as carbon suggests that the specimen underwent early diagenetic phosphatization. The presence of barite on top of the fossil was a later diagenetic event, during which barite was precipitated. Marine barite often forms in early diagenetic settings in association with decaying organic matter and mobile barium can precipitate as barite in sulfate-rich conditions, which the presence of iron and sulfur in the sediment indicate [66–69]. However, the early in the taphonomic process the barite formed remains uncertain.

The matrix of *Magnicornaspis garwoodi* gen. et sp. nov. is composed of aluminum, iron, magnesium, potassium, oxygen, and silicon, and limited sulfur, suggesting a likely composition of clay minerals, iron oxides, and potentially some pyrite.

### Rivière-du-Loup Formation as a potential Fossil-Lagerstätte

To date, there has been no formal documentation of the macrofossils from the Trois-Pistoles Group. Nevertheless, existing studies have documented the common presence of trilobites, graptolites, and brachiopods in some horizons [28, 40]. The addition of a corcoraniid here in the Rivière-du-Loup Formation provides interesting parallels with other sites worldwide, specifically the exceptional preservation seen in other Cambrian Fossil-Lagerstätten. While the majority of Furongian sequences globally appear to be dominated by biomineralized shelly faunas, the presence of non-mineralized arthropods in Quebec mirrors the Weeks Formation of Utah [70] and the Sandu Formation of South China [71]. This discovery is particularly significant in the context of the “Furongian Gap,” an evolutionary interregnum between the Cambrian Radiation and the Great Ordovician Biodiversification Event characterized by a marked drop in biodiversity [72–75]. Whether this gap represents a real biological decline driven by fluctuating environments, extreme climates, and widespread anoxia, or an apparent artifact of sampling failure and a lack of rock has been a core research question [76]. The occurrence of this specimen within the Appalachian margin suggests that these primitive arthropods maintained a cosmopolitan distribution during the final stages of the Cambrian, despite the prevailing “dearth of data” from this critical interval. Furthermore, finding this taxon in the Rivière-du-Loup Formation supports the view that the Furongian biodiversity signal has been significantly underestimated and a historical lack of intensive taxonomic study on deeper water lithofacies has led to an anthropogenic bias [77].

Likewise, the limited study of Corcoraniidae reflects the overall rarity of these fossils globally [1], a situation exacerbated by the small number of specimens typically available when material has been identified in collections (see Lerosey-Aubril, et al. [1]). The specimen described here does not substantially alleviate this issue. However, it does hint at the potential for exceptional preservation within Rivière-du-Loup Formation. Revisiting the type locality and conducting further investigation of the deposit may reveal another significant, late Cambrian site preserving soft-bodied organisms.

### Future for PaleoDEMs

Although PaleoDEMs are exceedingly important in inferring the geographic ranges of lesser-known fossil invertebrate taxa [50, 78], equally important is the incorporation of such reconstructions within Global Circulation Models (GCMs) to estimate the abiotic responses [75]. Over the past decade, advances in paleogeographic reconstruction and climate modeling have enabled paleoclimatologists to simulate global climate variables throughout the Phanerozoic using coupled ocean-atmosphere GCMs integrated with DEMs, atmospheric carbon dioxide concentrations (pCO₂), and solar luminosity estimates [79–81]. These models generate spatially explicit environmental raster datasets, including seasonal atmospheric and oceanic temperature and salinity that can be paired with paleo-occurrence data to construct paleoecological niche models (PaleoENMs). PaleoENMs are correlative models that estimate environmental suitability through deep time, and have become increasingly important for addressing ecological, evolutionary, and biogeographic questions [82–87]. Although the application of PaleoENMs to fossil taxa is a recent shift, they possess considerable potential to mitigate spatial and temporal gaps inherent in the fossil record. Further, these models can reconstruct past species distributions under changing climatic regimes [84].

The HadCM3 family of GCMs represents one of the most widely applied model suites for PaleoENM that has been used previously for terrestrial vertebrates and plants, including theropods, crocodilians, birds, and palms [88–93]. These applications have largely focused on abundant, well-preserved, and taxonomically well-resolved fossil groups, conditions that maximize model robustness. Invertebrate fossil groups, in many cases, meet or exceed these criteria. Taxa such as trilobites, bryozoans, brachiopods, and mollusks possess fossil records complete enough to explore large-scale patterns of evolutionary turnover, succession, and diversification [94–97]. Despite this richness, invertebrates remain underrepresented in PaleoENM studies, with only one study incorporating GCMs [98]. The extension of GCM-based PaleoENMs to invertebrate taxa represents a new frontier for examining macroecological and macroevolutionary dynamics, including patterns of persistence, habitat expansion/contraction, and ecological turnover across major climatic and geologic transitions.

In recent years, the centralization of large biodiversity datasets, such as the Global Biodiversity Information Facility (GBIF) and the Paleobiology Database, has enabled increasingly accurate and large-scale modeling of species’ ecological niches and geographic distributions. However, within PaleoENM, accurate representation of fossil species’ ranges remains limited by geological, preservational, taphonomic, and sampling biases that introduce downstream uncertainty in model construction [84, 98]. An additional limitation is the often-restricted sample size of fossil lineages within individual geologic stages, constraining models to static temporal slices and limiting the ability to reconstruct continuous shifts in species distributions through time. As a result, PaleoENMs frequently represent snapshots of environmental suitability rather than fully resolved temporal trajectories of ecological change.

Dynamic modeling approaches provide a framework for addressing these limitations by explicitly incorporating temporal variation in environmental conditions. During model construction, environmental variables and paleo-occurrence data are temporally matched within discrete time intervals, allowing models to be trained on time-specific species–environment relationships. These models are then projected onto equivalent environmental variables across successive time slices, enabling habitat suitability to be evaluated through changing climatic and paleogeographic conditions. By iteratively applying the same set of environmental predictors across multiple intervals, dynamic PaleoENMs allow changes in potential distributions to be interpreted as continuous environmental responses rather than as isolated reconstructions. Furthermore, this framework partially alleviates limitations associated with small sample sizes by allowing occurrences from across a lineage’s temporal range to inform the model. As such, dynamic approaches provide a means of approximating how species’ environmental tolerances and geographic ranges may have tracked long-term climatic and paleogeographic change through time.

## Conclusions

Here we present a new corcoraniid arthropod from the Furongian Rivière-du-Loup Formation and have explored the evolution, ecology, and preservation of this new material. The presence of hypertrophied cephalic spines in a Cambrian form suggests that these structures arose earlier than previously recognized. Palaeobiogeographic patterns illustrate broad, shallow-marine distributions for corcoraniids between the Cambrian and Ordovician, with minimal environmental turnover. Elemental analyses illustrate early diagenetic phosphatization with preserved carbonaceous material, followed by barite overprinting, which are common within Konservat-Lagerstätten-style preservation. The occurrence of this soft-bodied arthropod highlights the potential of the Rivière-du-Loup Formation as a rare, underexplored Konservat-Lagerstätte and supports the view that the “Furongian Gap” partly reflects sampling bias, rather than true biodiversity decline.

## Methods

### Imaging

The specimen is housed within the United States National Museum palaeontology collection (USNM PAL) under specimen number USNM PAL 801575 and was imaged at the American Museum of Natural History (AMNH) using an array of imaging techniques. The specimen was imaged under plain and flash photography using a Canon EOS 60D camera body and an EF 100 mm macro lens. It was also imaged using reflectance transformation imaging (RTI), with the goal of producing an interactive RTI image. To conduct the RTI, a Nikon Z 6II camera body was used with a Nikkor Z MC 105 mm macro lens. The images from the RTI dome were compiled into an interactive figure using ReLight Lab 2025 [99]. This produced Additional File 1: Fig. S1 that was uploaded into OSF at the following DOI: 10.17605/OSF.IO/GJ6BH.

### Systematic paleontology

When describing the specimen, we followed the systematic paleontology and descriptive terminology of Lerosey-Aubril et al. [1, 5] as these articles represent the most recent revision of the group.

### Reconstruction

The stipple reconstruction of the holotype was constructed using traditional media and techniques, including primarily fine liners. The final illustration was further modified within Microsoft PowerPoint prior to completion.

### Scanning electron microscopy

To examine the elemental composition of the specimen, a scanning electron microscope (SEM) coupled with energy-dispersive X-ray spectroscopy (EDS) was used. The specimen was analyzed uncoated under low-vacuum conditions at an accelerating voltage of 20 kV, a working distance of 6 mm, and a beam current of 16 nA, using a Hitachi S-4700 SEM at the AMNH to collect back-scattered electron (BSE) images. Elemental maps were acquired using a Bruker AXS Quantax 4010 detector at a resolution of 2560×1920 pixels.

### Paleogeographic reconstruction

Corcoraniidae paleogeographic distributions were generated for the Cambrian and Ordovician using occurrence records of corcoraniid observations from the PBDB (Downloaded October 2025, Additional File 2: Data Table 1). Due to limited records of the family (*n* = 23), all corcoraniid occurrences that could be verified in the literature were used. Time intervals were binned to the Cambrian (538.8–485.4 Mya; 19 occurrences), the Ordovician (485.4–443.8 Mya; four occurrences) (Additional File 2: Data Table 1). Paleo-environmental maps were derived using the Paleo-Digital Elevation Model (PaleoDEM) dataset from Scotese and Wright [49], selecting the 1° × 1° PALEOMAP dataset. PALEOMAP paleocoastlines were derived from the online Gplates portal [100] (https://gwsdoc.gplates.org/) using the R package *sf* [101]. Since PALEOMAP PaleoDEMs consist of three-million-year time slices that approximate geological stages, each corcoraniid occurrence was first assigned a representative age calculated as the midpoint between its minimum and maximum age bounds. Occurrences were then paleo-rotated to the closest PALEOMAP time slice to that midpoint age. For visualization purposes, paleo-rotated occurrences, and the new observation, were displayed on PALEOMAP reconstructions corresponding to the median age of each geological period (Cambrian: 520 Ma; Ordovician: 475 Ma) using QGIS v3.36.1 [102].

## Supplementary Information

Additional file 1. Fig S1: RTI image of USNM PAL 801575. Found at https://doi.org/10.17605/OSF.IO/GJ6BH

Additional file 2. Data Table 1: Data from the PBDB used for palaeobiogeographical analyses

## Data Availability

No datasets were generated or analysed during the current study.

## Abbreviations

AMNH: American Museum of Natural History
BSE: Back-scattered electron
EDS: Energy-dispersive X-ray spectroscopy
GBIF: Global Biodiversity Information Facility
GCM: Global Circulation Model
PaleoDEM: Paleo-Digital Elevation Model
PaleoENM: Paleoecological niche model
PBDB: Paleobiological Database
RTI: Reflectance transformation imaging
SEM: Scanning electron microscope
USNM PAL: United States National Museum palaeontology collection
